# Recurrent Cutaneous Pyogenic Granuloma of the Nose Following Rhinoplasty: A Case Report

**DOI:** 10.7759/cureus.21849

**Published:** 2022-02-02

**Authors:** Fareeda S Alghamdi Jr., Ghassan Barnawi, Awadh M Alamri, Hussam Alqarni, Sameer Kutbi

**Affiliations:** 1 Dermatology, King Saud Bin Abdulaziz University for Health Sciences College of Medicine, Jeddah, SAU; 2 Dermatology, King Abdulaziz Medical City, King Saud Bin Abdulaziz University for Health Sciences College of Medicine, Jeddah, SAU

**Keywords:** cosmetics, dermatology, rhinoplasty, granuloma, pyogenic

## Abstract

Pyogenic granuloma (PG) is a vascular tumor originating from the skin and mucosal membranes. The most common sites include the oral and nasal cavities. It appears as a solitary erythematous lesion that bleeds easily. Various triggers were present in the literature such as pregnancy, drugs, and trauma. Trauma to the nose such as nose piercing was reported several times. However, there has been only one case study that has reported rhinoplasty as a potential trigger for the development of PG. Here, we report a case of recurrent PG following rhinoplasty in a 45-year old female.

## Introduction

Pyogenic granuloma (PG) or lobular capillary hemangioma refers to a benign vascular tumor arising from the skin or mucosal membranes of the head and neck, and it commonly affects the oral and nasal cavities [1]. Clinically, it appears as a red to pink to purple solitary, lobulated, friable, and highly vascularized lesion with a smooth or exophytic surface [2]. Historically, PG was thought to be due to an infectious etiology hence the term “pyogenic” was given. However, the etiology of PG is not clearly understood and is still unknown to this day. Several factors were implicated in the pathogenesis of PG, including infections, trauma, pregnancy, and drugs [3]. Moreover, hormonal factors are suggested as predisposing factors to PG, as it is seen commonly during pregnancy and has a peak incidence at the childbearing age, especially when a positive history of hormonal contraceptive use is present [1]. The likely explanation behind trauma as a cause for PG is that insults may result in a disproportion between pro-angiogenic and anti-angiogenic factors leading to the proliferation of capillaries in a rapid fashion, consequently creating a friable and lobulated neovascularization [1].

A commonly reported trauma to the nose that led to PG formation was nose piercing [4]. While trauma to the nose plays a role in PG development, the literature has only reported one case of an infraorbital PG following rhinoplasty [5]. In rhinoplasty, referred to as a “nose job” by patients, the manipulation of nasal tissues creates some type of trauma to the nose. This procedure can be classified as primary and secondary, which is a rhinoplasty following an operation by another surgeon. Also, it can be classified according to the type of surgery performed such as closed and open rhinoplasty, osteotomies, septoplasty, cephalic trim, and altering tip projection [6].

Complications following rhinoplasty are not uncommon, and they range from systemic complications like deep venous thrombosis/pulmonary embolism to local complications to the nose including hematoma, infection, scarring, and olfactory changes [7]. Nevertheless, PG may occur as a rare complication of rhinoplasty. In the present case report, we report a novel case of cutaneous PG of the nose following rhinoplasty in a middle-aged woman.

## Case presentation

**Figure 1:**
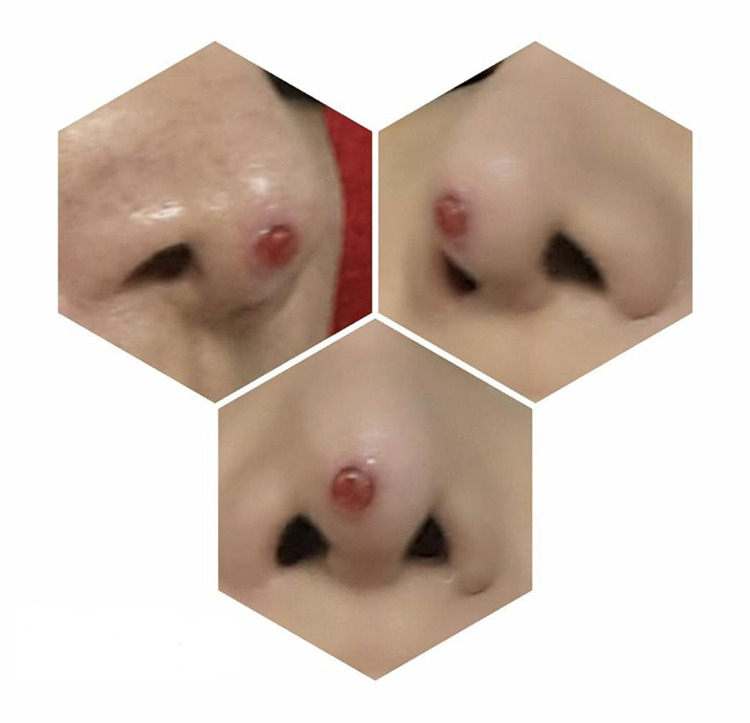
First Presentation

**Figure 2:**
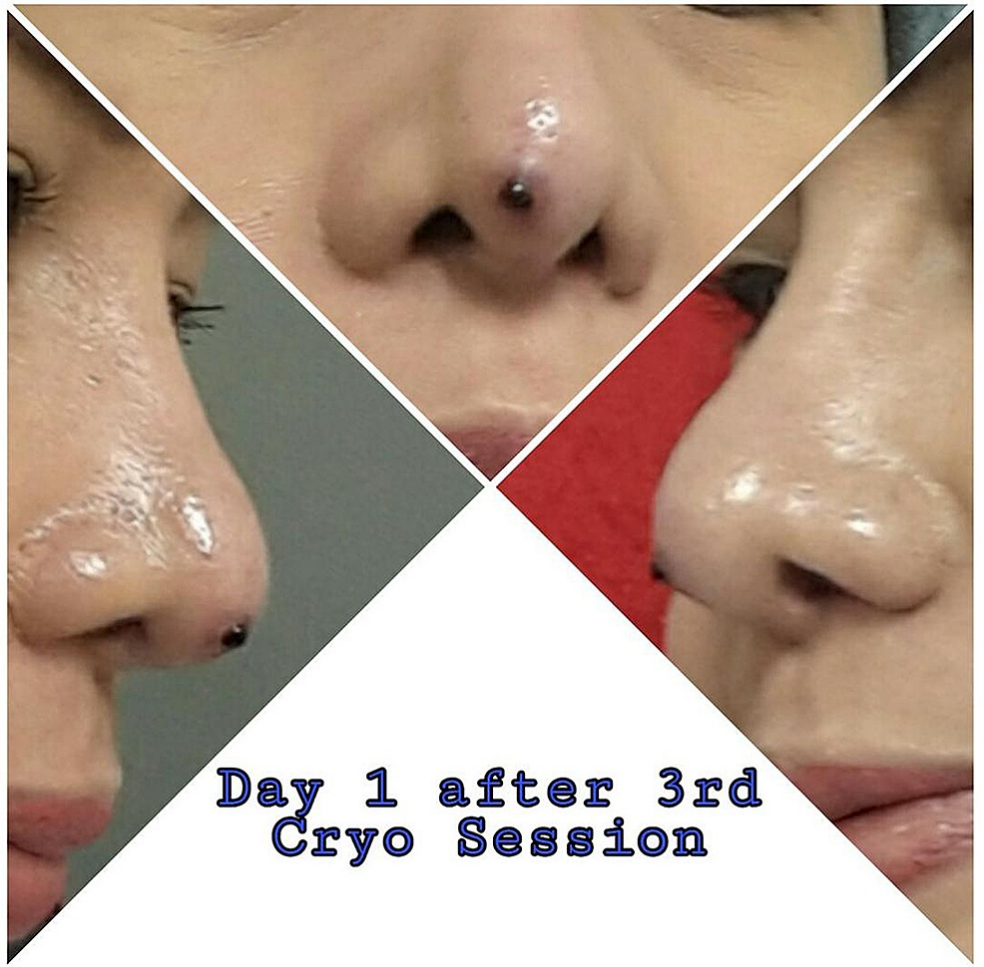
Day 1 After the Third Cryotherapy Session

A 45-year-old woman presented to our clinic in September 2019 with a red solitary friable papule at the tip of the nose that started to grow three months earlier (Figure 1). The lesion initially appeared in mid-June of 2019 as a pimple measuring 2-3 mm and evolved into a larger papule (5-6 mm) with occasional watery and bloody discharge. The patient had self-medicated with several over-the-shelf and generic topical treatments, including honey and antimicrobial ointments, but showed no improvement. The lesion continued to enlarge in size over time despite the use of the topicals by the patient. It was associated with discomfort and mild pain especially when pressure was applied to the area, leading to interference with the patient’s quality of life. Upon history, our patient reported undergoing a nasal augmentation (rhinoplasty) in 2018 with the use of expanded polytetrafluoroethylene (Gore-Tex) implants. No history of another trauma to the area or other family history of the same presentation. The clinical features of the lesion were typical for a pyogenic granuloma, and treatment was aimed to remove the lesion for definitive treatment and histopathological assessment. Due to the site of the lesion and possible cosmetic concerns, the patient underwent three sessions of cryotherapy on three consecutive days to achieve hemostasis and possible reduction in size in preparation for a shave excision with base cauterization (Figure 2).

**Figure 3:**
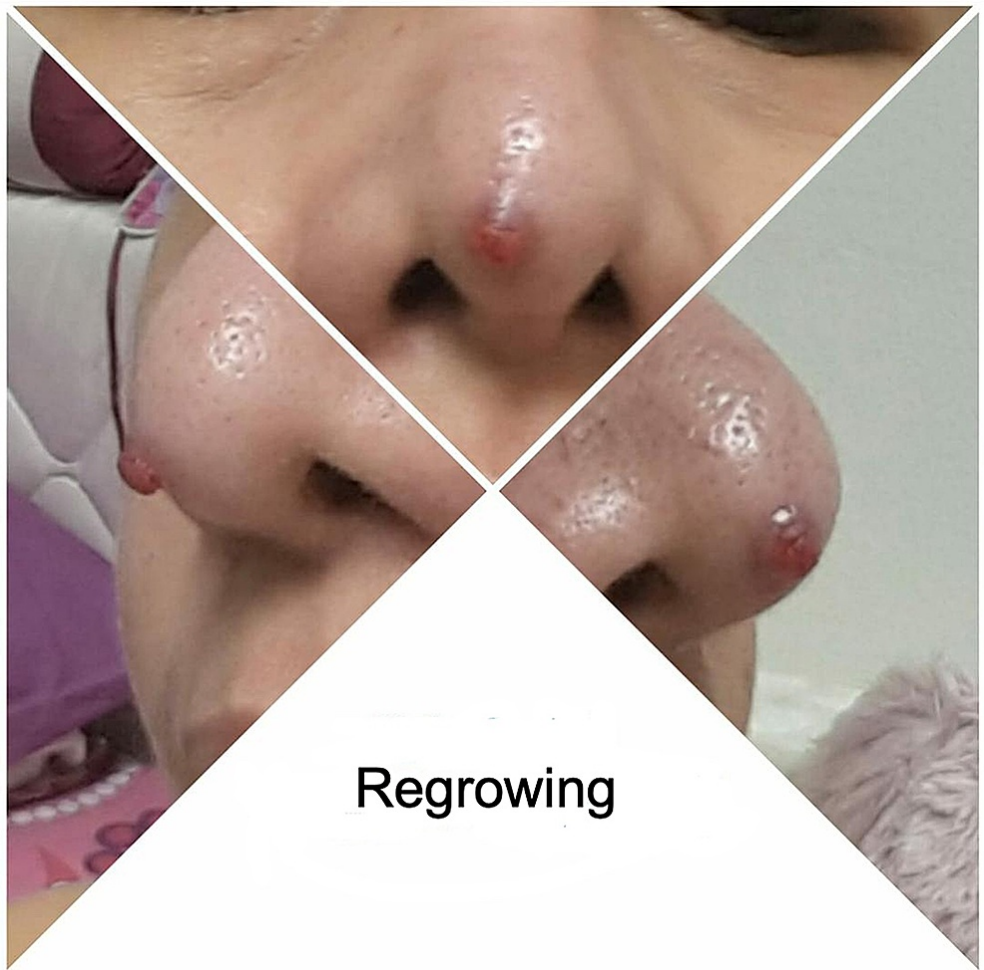
First Recurrence

**Figure 4:**
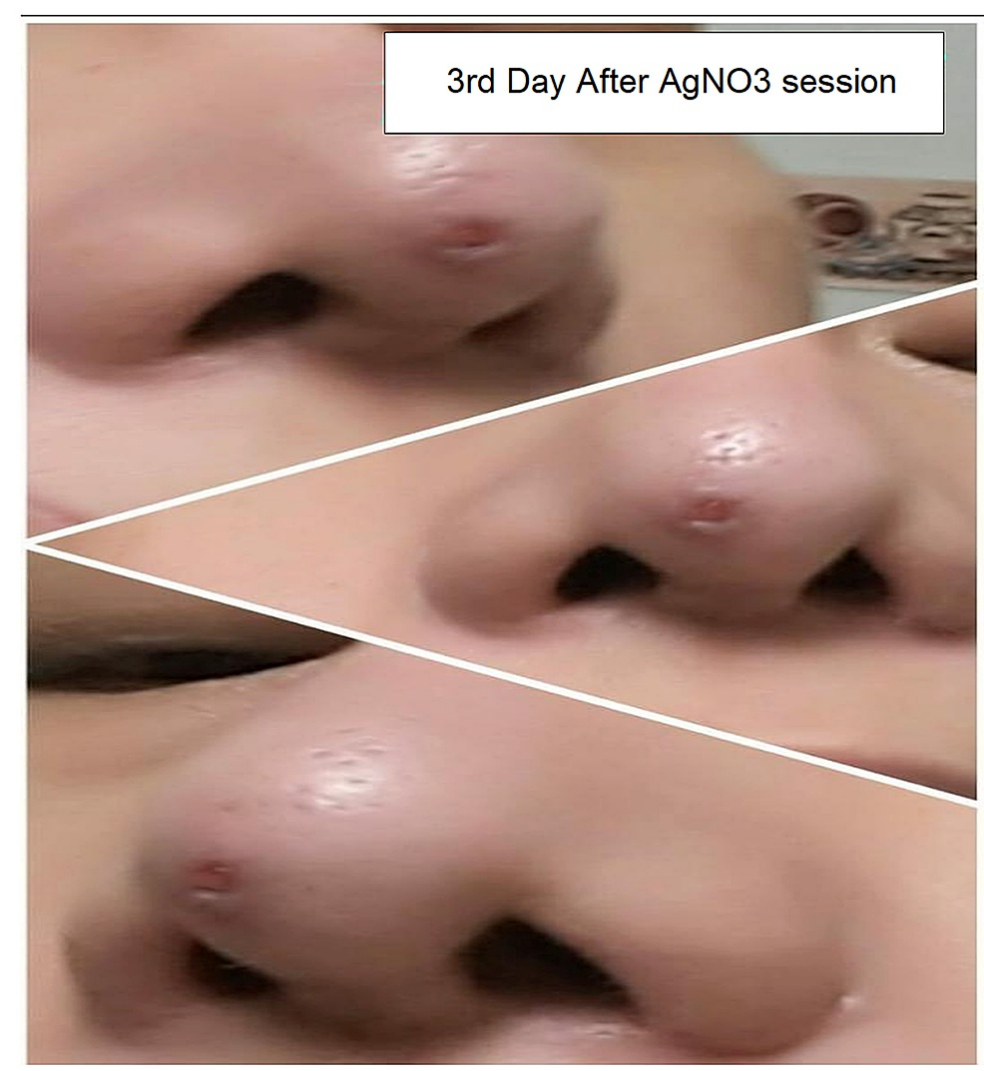
Healing After Silver Nitrate (AgNO3)

Shave excision with electrocauterization of the base of the lesion was performed, and microscopic analysis of the lesion showed a granulation tissue-like tumor with prominent vascular spaces and endothelial cells without cellular atypia, which was consistent with the diagnosis of a pyogenic granuloma. The lesion appeared to heal appropriately throughout the following days but started regrowing rapidly into a round red papule at the same site within a few weeks. The patient returned six weeks later with a cutaneous pink itchy papule at the nasal tip (Figure 3). The diagnosis was made clinically at the second time, as the lesion appeared quite similar to the patient's first presentation and histopathological assessment was not required. A different line of treatment was used for management at that time, and the patient was treated with three separate sessions of silver nitrate (AgNO_3_) cautery. The lesion demonstrated rapid regression in size after each treatment session and continued to heal after the last session with no signs of recurrence for the following three weeks (Figure 4).

Eight months later, however, the patient contracted coronavirus disease 2019 (COVID-19) infection and started to notice a small wound or ulcer-like lesion developing at the site of the previously treated pyogenic granuloma. The lesion started to grow very slowly again into a papular lesion over months. The patient was referred to a plastic surgeon for further assessment of the implant because local and systemic infections are considered one of the factors that may lead to the development of PG. In addition, an infected Gore-Tex implant is a rare complication of this type of implant, which necessitates implant removal. Our patient was assessed for the viability of the implant and to rule out any active association of the procedure with the patient’s recurrent presentations. The patient also underwent wide local excision of the lesion with primary closure. The procedure allowed for direct visualization of the implant and the area beneath the lesion, which showed no signs of infection. However, a deep extension of the tumor base into the subcutaneous tissue possibly due to initial incomplete excision, which eventually contributed to our patient's disease recurrence, was noticed. The site of the lesion has been healing since the procedure with no signs of recurrence so far.

## Discussion

**Figure 5:**
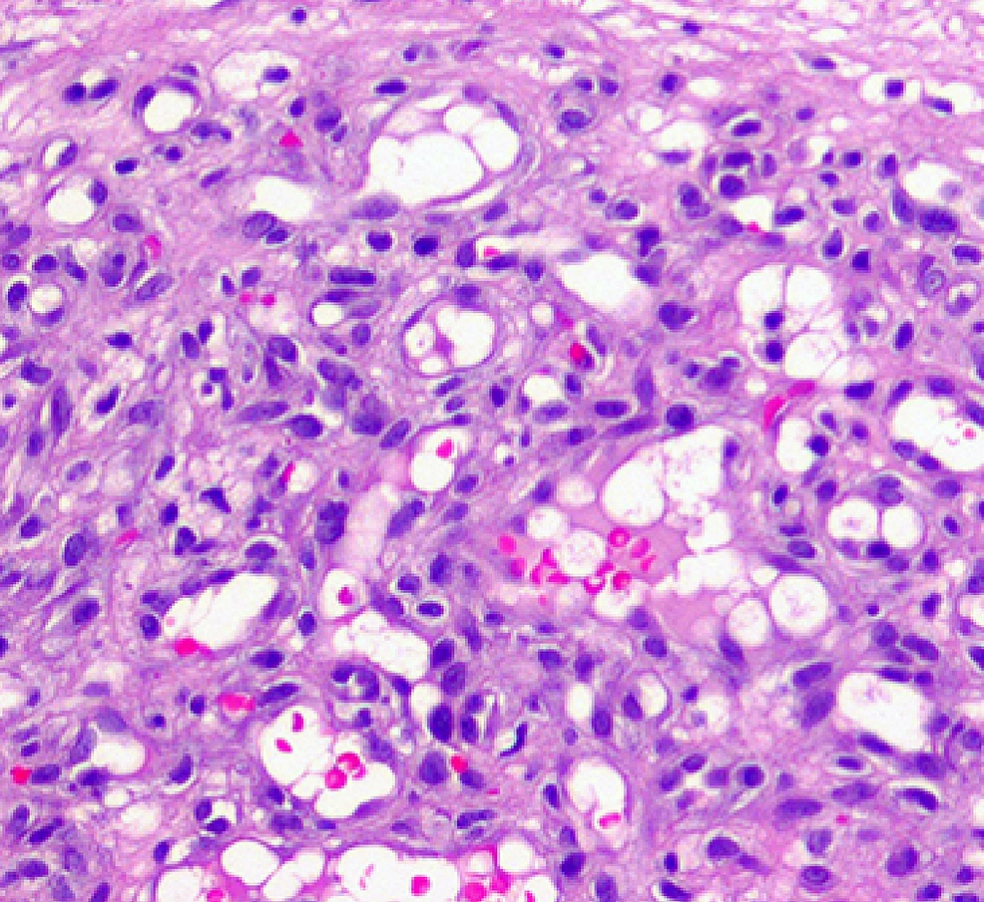
Pyogenic Granuloma Histopathology At higher power, the lesion consists of condensed small-caliber vessels with scattered red blood cells. The stroma is fibromyxoid with scattered inflammatory cells. Cytologic atypia and mitoses are not present.

Pyogenic granuloma is a benign vascular lesion of unknown etiology that affects the skin and mucous membranes. This vascular lesion tends to grow rapidly and reaches its final size within a few weeks. It can grow on the skin surface or other mucous membranes like the nasal cavity and usually presents as a painless pink or red solitary papule that bleeds easily upon trauma or irritation [1]. Despite its idiopathic nature, pyogenic granuloma has been associated most commonly with hormonal changes, physical trauma that disrupts the skin integrity, and systemic or local infections [1]. Pregnancy and oral contraceptives by far have been the two most common triggers for pyogenic granuloma related to hormonal changes. Trauma, on the other hand, has also been shown to be an underlying trigger for many cases of pyogenic granuloma, where the traumatized skin acts as a nidus for abnormal vascular proliferation. The diagnosis is usually done clinically when patients present with lesions that demonstrate classical gross features consistent with history [8]. Dermoscopic and microscopic features are usually assessed to confirm the diagnosis and rule out the possibility of other dermatologic lesions that may require further treatment such as basal cell carcinoma (BCC). Using dermoscopy, the absence of specific dermoscopic criteria for other skin tumors and a reddish homogeneous area surrounded by a white collarette are the most frequent dermoscopic patterns in pyogenic granuloma [9]. Histological patterns typically found in PG include an erosive or ulcerative epidermis overlying numerous capillaries and venules with plump endothelial cells arrayed radially toward the skin surface amidst an edematous stroma containing a mixed inflammatory infiltrate (Figure 5) [10-11]. The main goal in the treatment of PG is to identify the most likely underlying trigger. For example, medications that are commonly associated with hormonal changes, such as contraceptives, should be discussed with the patient to be stopped or changed to other effective contraceptive methods. Removal of a clear provoking traumatic event should also be done first if identified. Lesions that are likely caused by an underlying trigger may regress or completely resolve upon cessation of the trigger. If a lesion persists despite managing possible identified causes, or no clear triggers were identified, treatment of PG can be achieved surgically, medically, or combined. Several factors are considered when deciding on the type of treatment such as the location and size of the lesion and the patient’s age and preference [8]. Medical treatment is usually used for small lesions particularly those that present on the face and other exposed body surfaces where surgical excision is usually undesired by patients. Topical beta-blocker, imiquimod, and ingenol mebutate are all considered effective medical treatments for both cutaneous and mucosal PG [12-14]. Other non-surgical interventions that demonstrate comparable efficacy for small PG include cryotherapy and silver-nitrate cautery [15-16]. The two most common surgical techniques are either shave removal with electrocautery or surgical excision with primary closure. These two procedures are not only associated with a lower recurrence rate (10%-%15) particularly with incompletely excised lesions, compared to medical treatment but also provide a tissue sample for histologic confirmation [17-18]. Follow-up appointments for successfully treated pyogenic granuloma are not routinely advised and patients can live their lives with no concern. However, if any signs of recurrence are seen, patients should seek medical care as soon as possible for early treatment and management.

## Conclusions

Since the literature has only reported one case of PG following rhinoplasty, and the lesion was found in the infraorbital region, this case report presents a novel case of a recurrent cutaneous pyogenic granuloma of the nose following rhinoplasty. We found out that cutaneous PG of the nose can be a complication of this procedure, and some factors like infections and incomplete surgical excision of these lesions can lead to treatment failure or recurrence. We hope that this case draws more attention to considering PG as a potential complication for patients undergoing rhinoplasty. In addition, we recommend conducting more research that investigates the association between rhinoplasty and the occurrence of PG.
